# Diminished heart beat non-stationarities in congestive heart failure

**DOI:** 10.3389/fphys.2013.00107

**Published:** 2013-05-15

**Authors:** Sabrina Camargo, Maik Riedl, Celia Anteneodo, Jürgen Kurths, Niels Wessel

**Affiliations:** ^1^Department of Physics, Humboldt-Universität zu BerlinBerlin, Germany; ^2^Department of Physics, PUC-RioRio de Janeiro, Brazil; ^3^National Institute of Science and Technology for Complex SystemsRio de Janeiro, Brazil; ^4^Potsdam Institute for Climate Impact ResearchPotsdam, Germany; ^5^Institute for Complex Systems and Mathematical Biology, University of AberdeenAberdeen, UK

**Keywords:** heart rate variability, non-stationarity, segmentation

## Abstract

Studies on heart rate variability (HRV) have become popular and the possibility of diagnosis based on non-invasive techniques compels us to overcome the difficulties originated on the environmental changes that can affect the signal. We perform a non-parametric segmentation which consists of locating the points where the signal can be split into stationary segments. By finding stationary segments we are able to analyze the size of these segments and evaluate how the signal changes from one segment to another, looking at the statistical moments given in each patch, for example, mean and variance. We analyze HRV data for 15 patients with congestive heart failure (CHF; 11 males, 4 females, age 56±11 years), 18 elderly healthy subjects (EH; 11 males, 7 females, age 50±7 years), and 15 young healthy subjects (YH; 11 females, 4 males, age 31±6 years). Our results confirm higher variance for YH, and EH, while CHF displays diminished variance with *p*-values <0.01, when compared to the healthy groups, presenting higher HRV in healthy subjects. Moreover, it is possible to distinguish between YH and EH with *p* < 0.05 through the segmentation outcomes. We found high correlations between the results of segmentation and standard measures of HRV analysis and a connection to results of detrended fluctuation analysis (DFA). The segmentation applied to HRV studies detects aging and pathological conditions effects on the non-stationary behavior of the analyzed groups, promising to contribute in complexity analysis and providing risk stratification measures.

## 1. Introduction

It has been a long time since studies on heart rate variability (HRV) have become as popular as the many devices available to record the cardiac and autonomic activity (Akselrod et al., 1985). HRV refers to heart beating signal itself as well to the time between consecutive heart beatings. As reported in literature, several diseases and conditions, as myocardial infarction, diabetic neuropathy, and myocardial dysfunction, for example, are related to decreasing HRV (Pagani et al., 1988; La Rovere et al., 1998), pointing to a connection between healthiness and complexity. Also, the effects of aging are known to present higher HRV in younger individuals, compared to elderly ones (Task Force, 1996). The possibility of diagnosis based on non-invasive techniques such as HRV studies is widely investigated (Kurths et al., 1995) and compels us to overcome the difficulties originated on the environmental changes that can affect the signal, eventually pointing out to a false disruption of the dynamics or leading to neglect a real one. Besides environmental conditions, non-stationarities can arise from multiple regulatory mechanisms, for example blood flow and respiration, operating concomitantly and varying over time, with each subsystem presenting its own time scale (Porta et al., 2009), and since the activation of only one control mechanism is not possible, the cardiac record will carry non-stationarities related to the superposition of different regulatory mechanisms (Magagnin et al., 2011). Spectral analysis, for example, relies on the assumption of weak stationarity, where the mean value is constant and the covariance is only dependent on a time shift, but even in controlled environments, it is questionable whether the efficiency of such control ensures these conditions.

The idea of the segmentation applied to time series is to provide patches of the signal where stationarity is verified. Bernaola-Galván et al. (2001) introduced a mean based segmentation algorithm which looks for points along the signal where the difference of the mean of the resultant segments is maximal. Since the mean based segmentation has *t*-statistics computed for equal variance samples as the criterion for locating the cutting points, no information about the variances could be inferred. Instead of testing the difference for the mean, we perform a non-parametric segmentation (Camargo et al., 2011), taking into account the whole distribution, with all moments, e.g., mean and variance. In the case of HRV, the known amplitude-frequency coupling of the dominant short-term oscillation, the respiratory sinus-arrhythmia, connects the changes in the variance with the time-dependent covariance, implying non-stationarity (Hirsch and Bishop, 1981).

## 2. Materials and methods

For analysis, we consider 24 h measurements of the electrocardiogram of three groups consisting of young and elderly subjects, and patients suffering from congestive heart failure. The groups are composed of 15 patients with congestive heart failure (CHF; 11 males, 4 females, age 56±11 years), 18 elderly healthy subjects (EH; 11 males, 7 females, age 50±7 years), and 15 young healthy subjects (YH; 11 females, 4 males, age 31±6 years). Data for the CHF patients and the YH subjects are available from PhysioNet (Goldberger et al., 2000). Data for the EH were provided by the Charité in Berlin (Wessel et al., 2003). The series of time intervals between consecutive heart beats, the beat-to-beat intervals, are extracted from the electrocardiograms (Suhrbier et al., 2006). All resultant signals were filtered in order to avoid ectopic beats (Wessel et al., 2007).

We have chosen a non-parametric method based on the comparison of the two segments resulting from a hypothetical cutting, by building the empirical cumulative distribution of the points to the left and to the right of this cut and looking for the cut which would provide the maximal distance between the empirical cumulative distributions, namely the Kolmogorov–Smirnov distance. After determining the position of the cut, we check for its statistical significance, and if necessary, we check for a minimum length. If the required critical distance value, for a given significance level, and the minimum length are verified, then the signal is split into two segments, that on their turns, will be examined in order to find other cutting points. Segmentation ends when no significant difference is found or when the remaining segment is smaller than the minimum. The segmentation procedure can be summarized as follows: given a time series, {*x*_*i*_, *i*_1_ ≤ *i* ≤ *i*_*n*_}, a sliding pointer is moved from *i* = *i*_1_, …, *i*_*n* − 1_ in order to compare the two fragments of sizes *n*_*L*_ = *i*_*p*_ − *i*_1_ + 1, and *n*_R_ = *i*_*n*_ − *i*_*p*_, where *i*_*p*_ is the position of the pointer. Then one selects the position *i*_max_ that maximizes the Kolmogorov–Smirnov (KS) statistics

(1) $$D\equiv D_{\text{KS}}{\left(1/n_{L}+1/n_{R}\right)}^{-1/2},$$

where *D*_KS_ is the maximal distance between the cumulative distributions of the samples. After determining the position *i*_max_ of the maximal distance *D*, *D*^max^, one checks statistical significance (at a chosen significance level α = 1 − *P*_0_) of a potentially relevant cut at *i*_max_ by comparison with the result that would be obtained was the sequence random. The critical value is given by the empirical expression

(2) $$D_{\text{crit}}^{\text{max}}(n)=a{\left(\ln n-b\right)}^{c},$$

(*a*, *b*, *c*) = (1.52, 1.80, 0.14) for *P*_0_ = 0.95, *n* = *n*_*L*_ + *n*_*R*_. Equation (2) was obtained by determining *D*^max^ of a large number of sequences of *n* independent and identically distributed Gaussian numbers, and then, using the complementary cumulative distribution of *D*^max^, we found the critical values for each significance level (Camargo et al., 2011).

**Figure 1:**
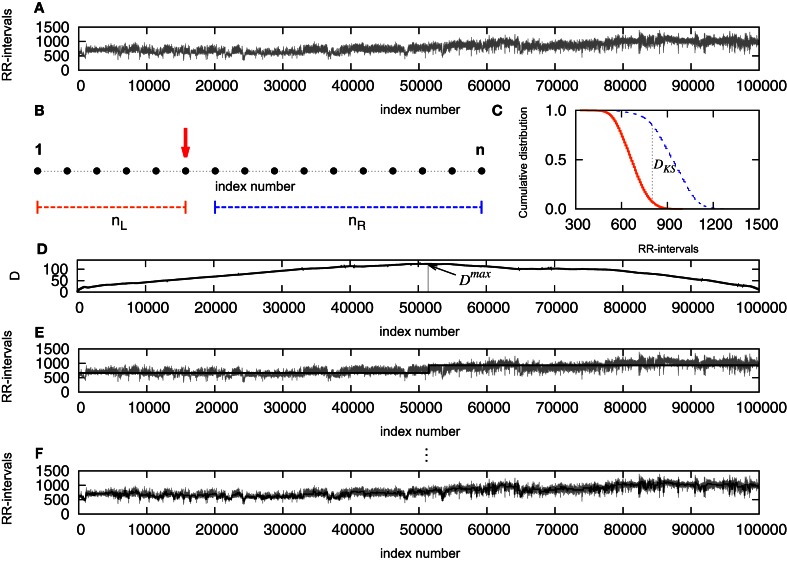
**(A)** Original signal, that will be examined point to point, as indicated in **(B)**, splitting the signal into two parts and computing the distance between the empirical cumulative distribution of each side **(C)**. Once this step is completed, one will have a distance *D* associated to each point of the signal **(D)** and the point where *D* is maximal is determined. The empirical expression given by Equation (2) is used to verify the significance level of the cut, and if *D* exceeds *D*_crit_ and the segment results not smaller than the minimum, the signal is cut. As a result, the signal is split into two segments **(E)**. Repeating the procedure, for each new segment resulting from a cut, until no significant differences found or the length reaches a value smaller than the minimum, one will have the signal fragmented in segments **(F)**.

The potential cut ticks the first stage if *D*^max^ exceeds its critical value for the selected significance level *D*^max^_crit_(*n*). If each resulting segment is greater than a defined minimum ℓ_0_, then the pointer is set and the procedure is recursively applied starting from the left patches until no patch is segmented. See Camargo et al. (2011) for further details. We performed the KS-segmentation with ℓ_0_ = 30 and *P*_0_ = 0.95. This value of ℓ_0_ is chosen in correspondence to the defined higher edge frequency of the very low frequency (VLF) band of heart rate with 0.03 Hz (Task Force, 1996) providing at least a half period of this frequency in each segment. Figure 1 presents a schematic first step of the segmentation: in **(A)** we see the original signal, that will be examined point to point, as indicated in **(B)**, splitting the signal into two parts and computing the distance between the empirical cumulative distribution of each side **(C)**. Once this step is completed, one will have a distance *D* associated to each point of the signal **(D)** and we look then to the point where *D* is maximal. We verify the significance of this cut by the comparison with the critical value, and the segment minimum length required, then the signal is cut. As a result, we have the signal split into two segments **(E)**. Repeating the procedure, for each new segment resulting from a cut, until no significant differences found or the length reaches a value smaller than the minimum, one will have the signal fragmented in segments **(F)**.

## 3. Results

**Figure 2:**
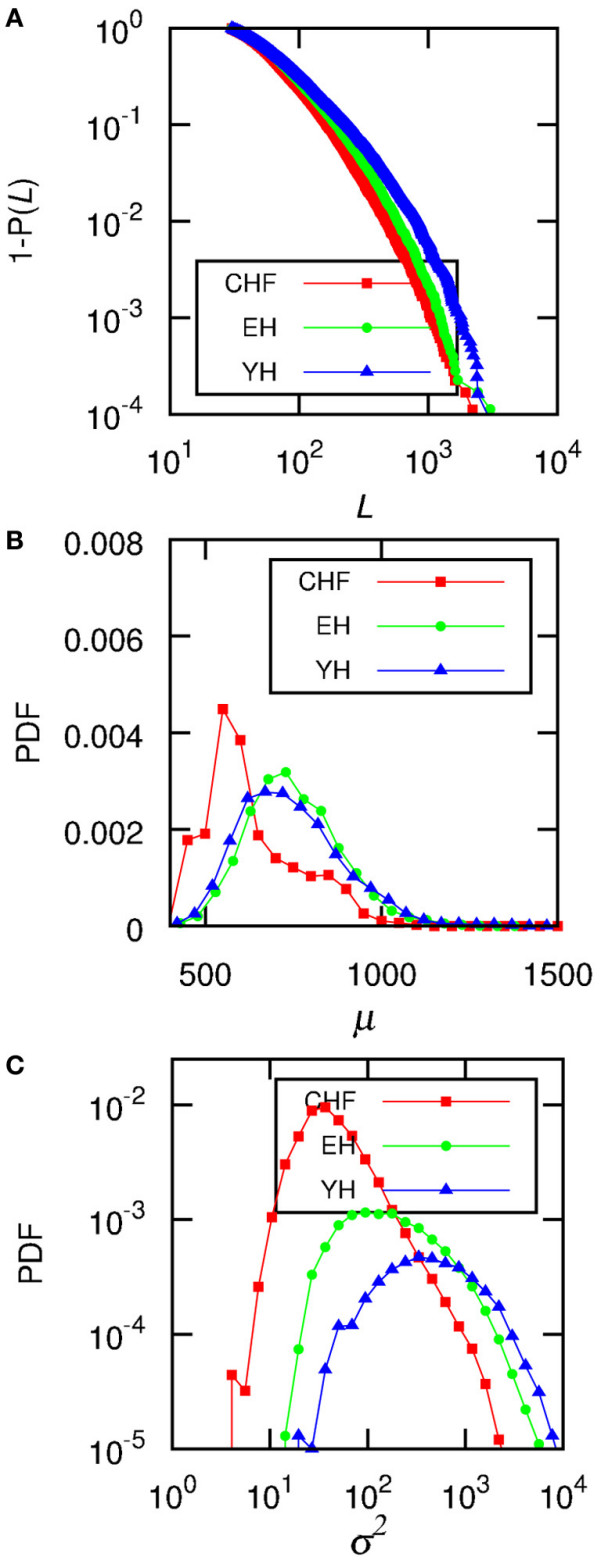
The data for all the subjects from each group were accumulated and **(A)** shows the complementary cumulative distributions of the size of each segment, *L*, **(B)** shows the distributions of the mean RR-interval, μ, of each segment, and **(C)** displays the distributions of the variance, σ^2^. The level of significance is *P*_0_ = 0.95, and the minimum segment size is ℓ_0_ = 30.

By finding stationary segments we are able to analyze the size of these segments and evaluate how the signal changes from one segment to another, looking at the statistical moments given in each patch, for example, mean and variance, in what we can call the intrinsic time scale of the signal (in opposition to arbitrary divisions of the signal). To quantify the non-stationarity, we obtain the length of the segments, *L*, the mean RR interval in each segment, μ, and the variance, σ^2^, associated to each segment, as well as the distributed jump size. jumps Figure 2 displays results of the accumulated data from all the subjects in each one of the three groups. In **(A)** we show the empirical cumulative distribution of the length of the segments *L*, **(B)** shows the distributions of the means of each segment, μ, and **(C)** the distributions of the variances σ^2^.

**Figure 3:**
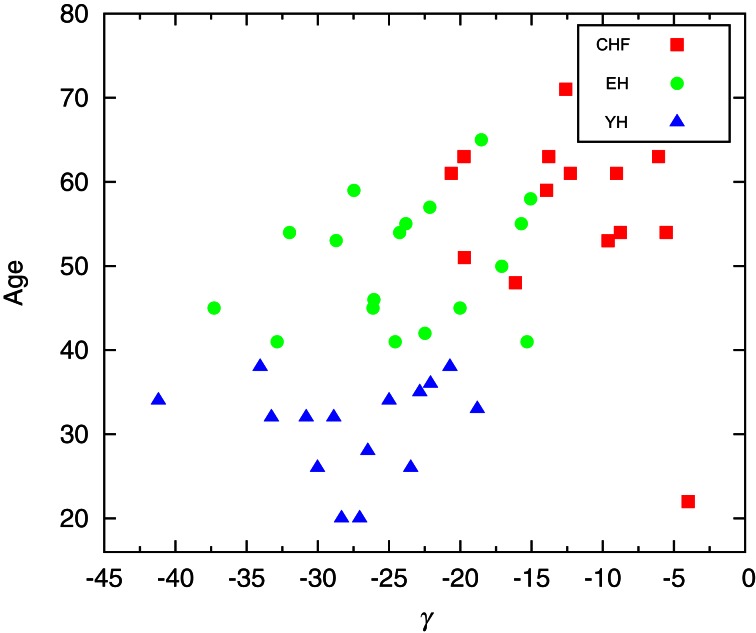
Scatter plot of the parameter giving the best fit of log[*PDF*(σ^2^)] = α[log(σ^2^)]^2^ + β[log(σ^2^)] + γ. Performing a Mann–Whitney U test for γ a *p*-value smaller than 10^-4^ is obtained when considering groups CHF–YH and CHF–EH, and *p* < 0.05 for EH–YH.

When considering individual distributions of the segment sizes, the empirical cumulative distribution of segment sizes is described by the double exponential curve *Ae*^−(*L*−ℓ_0_)/*L*_1_^ + (1 − *A*)*e*^−(*L*−ℓ_0_)/*L*_2_^. The Mann–Whitney U test for *L*_2_ provides significant differences between CHF and YH (*p* < 0.01) as well as CHF and EH (*p* < 0.05), pointing to differences in the occurrences of large segments between the pathological group and the healthy ones, while the occurrence of short segments shows no significant differences. Comparing the means of the segments does not improve the basic comparison of the means of the complete signal, thus the importance of accessing the second and further moments, if desirable, of each segment. Looking at the distribution of σ^2^ of each individual, we were able to fit the curve log[*PDF*(σ^2^)] = α[log(σ^2^)]^2^ + β[log(σ^2^)] + γ, aiming at individual parameters capable of distinguishing the groups. Performing a Mann–Whitney U test for γ values showed in Figure 3 we obtain *p* < 10^−4^ when considering groups CHF–YH and CHF–EH, and *p* < 0.05 for EH–YH. Although significant differences were found between CHF and the healthy groups when comparing means, the aging effect in the healthy groups is harder to detect, being pointed through symbolic dynamics POLVAR20 (Wessel et al., 2007) and now through the distribution of variances.

**Figure 4:**
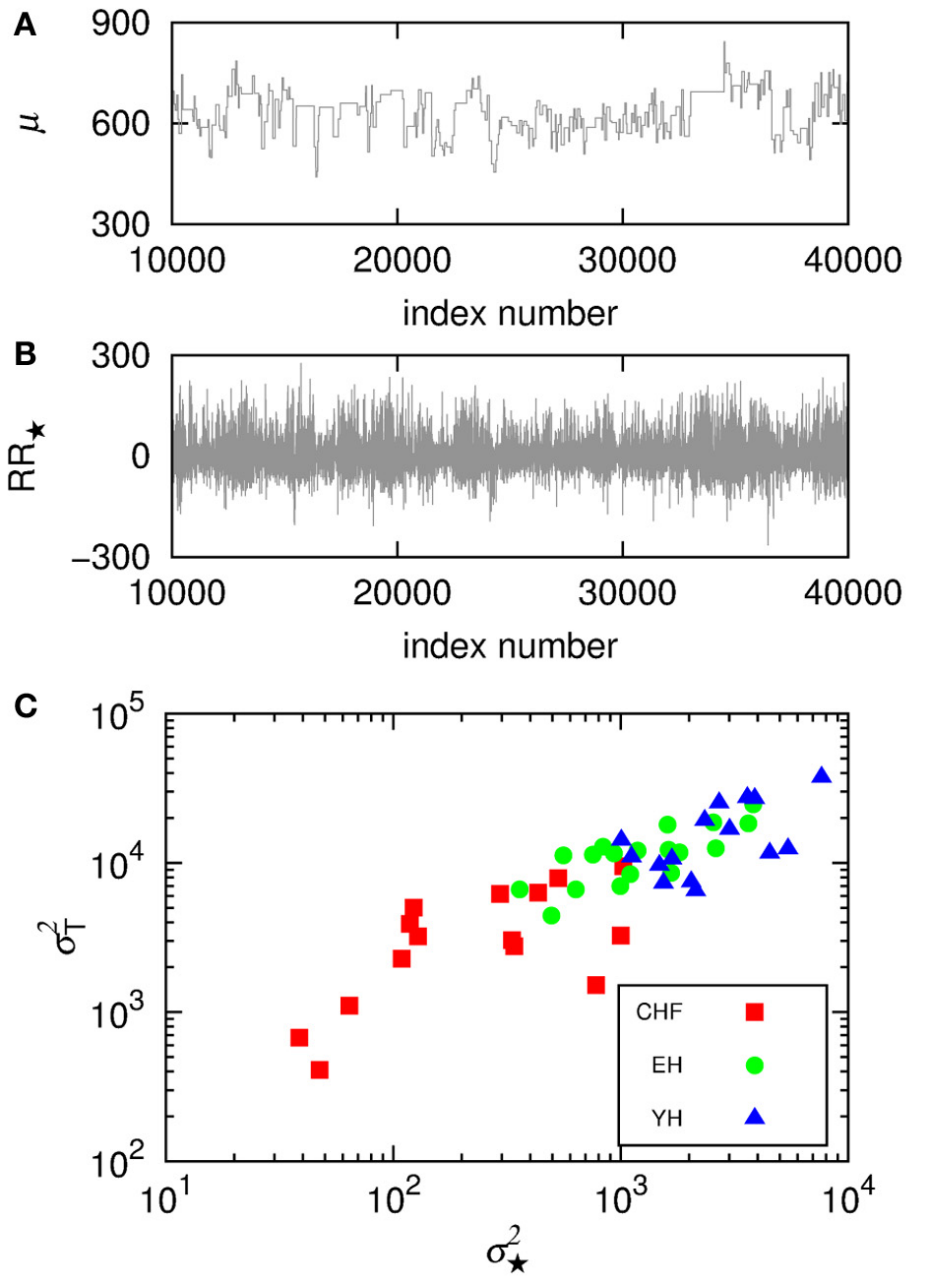
**(A)** Shows the trends given by the mean in the segments, μ, for a section of the segmented signal and **(B)** shows the signal *RR*_⋆_ resulting from the subtraction of the mean in each segment, μ. Then, **(C)** displays the variance in the detrended signal, σ^2^_⋆_, against the variance of the means, σ^2^_*T*_, for each subject.

Since it is usual to work with signals subtracted their mean, we compare how the signal subtracted by the segments mean, *RR*_⋆_, can improve the understanding of the non-stationary behavior of the groups. Figure 4A shows the trends given by the mean in the segments, μ for a section of the segmented signal and in **(B)** we see the signal *RR*_⋆_ resulting from the subtraction of the mean in each segment, μ. Then, we plot in **(C)** the variance in the detrended signal, σ^2^_⋆_, against the variance of μ, σ^2^_*T*_, for each subject. The results show correlations between the variability of the trends and the variability of the detrended signals, still displaying higher variability for healthy subjects, yet not providing differentiation due to aging effects.

**Figure 5:**
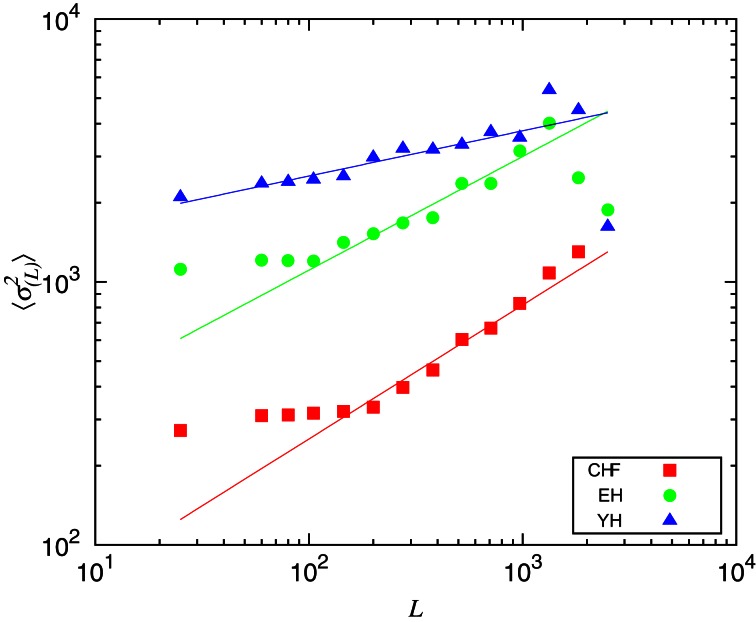
Mean variance of the segments 〈σ^2^_*L*_〉 given the segments length, *L*. The scaling exponents were obtained through numerical fittings, indicated by solid lines, with slopes ξ_CHF_ = 0.6, ξ_EH_ = 0.4, and ξ_YH_ = 0.2.

In order to understand the non-stationary behavior of the groups, we compute the mean variance of the segments 〈σ^2^_(*L*)_〉 given a segment length, *L*, displayed in Figure 5. One can see that CHF always presents the smaller values of 〈σ^2^_(*L*)_〉, while YH and EH systematically have higher values. It is worth to mention here the similarity of Figure 5 with plots given by detrended fluctuation analysis (DFA) (Peng et al., 1995), where the signal is integrated and then linearly detrended according to fixed sized boxes and then one verifies how the fluctuations behave with varying box sizes. With the segmentation, we have the box sizes replaced by the segments sizes and the fluctuations are given by the mean variances of the segments with given lengths. Computing the scaling exponents, obtained through the numerical fittings indicated in Figure 5 by solid lines, we obtain the slopes ξ_CHF_ = 0.6, ξ_EH_ = 0.4, and ξ_YH_ = 0.2. Results from DFA have shown that, in the range of 10^2^ ~ 10^4^ beats, the scaling exponent of the pathological group approaches to 1.5, corresponding to Brownian noise, that is integrated white noise. For random walk, DFA provides a scaling exponent of 0.5. In our segmentation analysis, as we do not integrate the signal, the correspondent pathological scaling exponent ξ_CHF_ = 0.6 indicates that random walk fluctuations dominate the dynamics of this group, in good accordance with DFA interpretation. For the EH and YH groups, the exponents ξ_EH_ = 0.4, and ξ_YH_ = 0.2 indicate power law correlations associated to the interchange of large and small RR intervals. In this sense, we can confirm the loss of complexity due to pathological condition and aging.

**Figure 6:**
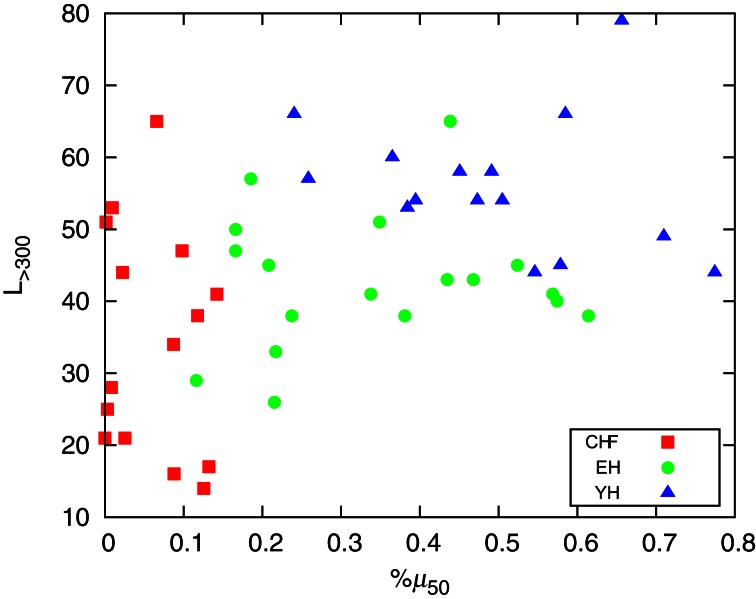
Plot of the number of segments with *L* > 300 (corresponding to approximately 5 min) and %μ_50_, the percentage of differences of the mean of two consecutive segments |μ_*i* + 1_ − μ_*i*_| > 50 ms.

To the extent of standard HRV analysis, in Figure 6 we plot the number of segments with *L* > 300, *L*_> 300_, corresponding to approximately 5 min, the shortest required segment length of HRV analysis (Task Force, 1996) and %μ_50_, the percentage of differences of the mean of two consecutive segments |μ_*i* + 1_ − μ_*i*_| > 50 ms in an analogy to the standard HRV measure pNN50, the percentage of consecutive RR intervals differing by more than 50 ms (Task Force, 1996). Figure 6 reveals that the pathological group, CHF, presents smaller %μ_50_ than the healthy groups EH and YH, with the last exhibiting also higher number of long segments. The aging affects the occurrence of larger segments as we know that the oldest subjects of EH are clustered around *L*_> 300_ = 40 and the oldest patients of CHF group are clustered around *L*_> 300_ = 30. We found high positive correlations between %μ_50_ and standard deviation of the signal, *sdNN*, as well as a negative correlation between *L*_> 300_ and the normalized very low frequency power, *VLF*/*P*, linking the segmentation outcomes to standard measures. The first one shows that *sdNN* is dominated by the large jumps whereas the second correlation reflects the reduction of very low frequencies for longer segments. For the pathological group, CHF, the correlation between *sdNN* and %μ_50_ is 0.94, while healthy groups, YH and EH, present a correlation of 0.90. For *VLF*/*P* and *L*_> 300_ we have a correlation of −0.77 for CHF and −0.81 for YH and EH.

## 4. Discussion

In this paper we present the analysis of non-stationarities in heart rate by means of a non-parametric segmentation algorithm, being able to detect significant differences between CHF and age-matched EH, as well as CHF and YH. Also, significant differences between YH and EH can be detected, showing the aging effect in the loss of complexity of the heart rate. We found a correspondence between DFA results, suggesting that random walk fluctuations dominate the dynamics of the pathological group. For the healthy groups, EH and YH, the long term correlations in the interchange of large and small RR intervals appears to be larger for EH and smaller for YH, denoting diminished non-stationarities due also to aging and pathological condition. Although KS-segmentation is limited to long term observations, since short data provide poor statistics of the segments, when considering previous segmentation methods, this choice for using the Kolmogorov–Smirnov distance instead of the mean of the segments provides the possibility of characterizing all the statistical moments of the segments, particularly the changes in the variance. According to our results, comparing means or standard deviation is not efficient to distinguish EH and YH, but the aging effect can be indicated by the variance in the segments. The aging effect has been detected when applying POLVAR20 (Wessel et al., 2003), which gives a good classification of the groups; however, no information about the dynamics underlying the systems is provided. Additionally, this segmentation procedure differs from arbitrarily splitting the signal in equal size segments and testing whether they come from the same distribution or not, rather it is the test for significant differences that determines the lengths of segments.

In terms of standard HRV analysis, a high positive correlation between %μ_50_ and *sdNN* shows that the standard deviation is dominated by the large jumps. A negative correlation between *L*_> 300_ and *VLF*/*P* reflects the reduction of very low frequencies for longer segments. Periodic breathing is prevalent in 30–50% of the patients with CHF (Hall et al., 1996; Lorenzi-Filho et al., 2005), and it has been reported (Lorenzi-Filho et al., 1999) that periodic breathing leads to modulation of the RR intervals in the VLF band, what would cause the algorithm to detect a larger number of short segments for CHF.

Through the outcomes of segmentation we have access to time characteristics of the signal that were no longer available, making possible a different approach to quantify non-stationarities in HRV analysis. Results are in agreement with previous knowledge and do not require arbitrary thresholds or exclude fragments of the time series. The individual risk stratification ability of this method relies on further applications to cardiological data bases.

### Conflict of interest statement

The authors declare that the research was conducted in the absence of any commercial or financial relationships that could be construed as a potential conflict of interest.
